# Towards automated manufacturing of clinical scale gene-modified T cells

**DOI:** 10.1186/2051-1426-2-S3-P21

**Published:** 2014-11-06

**Authors:** Katharina Drechsel, Daniela Mauer, Nadine Mockel-Tenbrinck, Constanze Lehmann, Hermann Bohnenkamp, Volker Huppert, Mario Assenmacher, Ian Johnston, Andrew Kaiser

**Affiliations:** 1 Miltenyi Biotec, Bergisch Gladbach, Germany

## 

Adoptive immunotherapy using gene-modified T cells redirected against cancer has proven clinical efficacy and tremendous potential in several medical fields. However, such personalized medicine faces several challenges in the complexity associated with the current clinical manufacturing methods, which hampers dissemination.

Conventionally, the preparation of autologous gene-modified T cells comprises many (open) handling steps, is labor intensive and is not adapted to treat large numbers of patients or for commercial manufacturing. Moreover, the cell-manufacturing process requires extensive training of personnel as well as a dedicated infrastructure, which restricts these clinical procedures to very few institutions worldwide. In order to face these challenges, Miltenyi Biotec has dedicated large efforts to further enable automation of cell manufacturing by developing a unique cell processing platform, the CliniMACS^® ^Prodigy, which enables the automated manufacturing of clinical grade gene-modified T cells in a closed single-use tubing set.

Starting from leukapheresis or whole blood products, the automated process enables magnetic labeling and enrichment of T cells, their subsequent stimulation, gene-modification with lentiviral vectors, expansion and final formulation with minimal user interaction. Within the process a novel stimulatory reagent has been implemented: MACS GMP TransAct™ in combination with TexMACS GMP Medium. TransAct is a colloidal reagent developed for polyclonal T cell stimulation that is soluble and can be removed by washing. The reagent is biodegradable, sterile filtered, and suitable for potent T cell activation, gene-modification, and expansion. Clinically relevant numbers of functional gene-modified T cells (>10^9^) have been generated within 10-14 days using the automated manufacturing process.

The flexibility and ease-of-use associated with this device and the developed process for clinical scale production of engineered T cells creates a solution for the treatment of large patient groups and facilitates economic commercial-scale manufacturing.

